# Imminent risk of COVID-19 in diabetes mellitus and undiagnosed diabetes mellitus patients

**DOI:** 10.11604/pamj.2020.36.158.24011

**Published:** 2020-07-06

**Authors:** Jyoti Elgiva John, Nitin Ashok John

**Affiliations:** 1 Department of Biochemistry, All India Institute of Medical Sciences, Nagpur, India,; 2 Department of Physiology, Dr Ram Manohar Lohia Institute of Medical Sciences, Lucknow, India

**Keywords:** Diabetes mellitus, undiagnosed diabetes mellitus, COVID-19, risk, Africa

## Abstract

Diabetes mellitus is a non-infectious disease and has affected about 425 million adults globally and nearly 15.9 million of them reside in Africa. Moreover, the prevalence of undiagnosed diabetes mellitus is very high in Africa and approximates to around 62%. Nearly 75% of the total deaths due to diabetes are in individuals lesser than 60 years of age. The multifaceted disease of diabetes mellitus produces chronic complications such as, neuropathy, nephropathy, retinopathy, microangiopathy etc. These patients of diabetes mellitus are more susceptible to infections due to compromised immune system. Hence these patients of diabetes mellitus and undiagnosed diabetes mellitus are at greater risk of contracting COVID-19 infections. The dual impact of pathophysiology of COVID-19 infections in diabetes mellitus may increase morbidity and mortality in these patients. Hence there is need of health awareness in diabetics as well in the high-risk group for diabetes such as persons with hypertension and obesity. The scarcity of health resources, shortage of trained medical personnel and disease burden of infectious and non-infectious diseases has laid a heavy impact on the economy in Africa and this has been further strained due to the COVID-19 pandemic. The practice of preventive measures by the risk group of Undiagnosed Diabetes Mellitus patients will prevent them from getting infected by COVID-19 and at the same time decrease mortality rates and hence the undiscovered group that is the patients of undiagnosed diabetes mellitus needs to be vigilant regarding safe preventive practices.

## Commentary

Diabetes Mellitus (DM) is the chronic non communicable disease affecting millions worldwide. Diabetes which was once known as disease of elite earlier has now impacted the general population from all socioeconomic strata because of the increasing stress and sedentary life style eventually leading to obesity and obesity associated complications such as hypertension and Type II Diabetes Mellitus. The disease if remains undiagnosed may lead to complications such as neuropathy, nephropathy, micro- and macro-angiopathies, and retinopathy [1]. Moreover the patients of diabetes mellitus are more susceptible to infections and are at risk of developing infectious diseases such as tuberculosis and pneumonia as well as non-infectious disease such as cardiovascular and kidney diseases. The prevalence of infectious diseases is higher in patients of DM. The probable causes of increased susceptibility to infections in patients of DM is attributed to the fact that the persistent hyperglycemia in patients of DM enhances the virulent activity of few pathogens; decreased chemotaxis and phagocytic activities of phagocyte cells, decreased interleukins production on exposure to antigens and loss of mobilization characteristics of leukocytes. DM may also lead to metabolic complications such as hypoglycemia, ketoacidosis, and coma [1].

The total number of patients of DM globally as per the data of 2017 released by International Diabetes Federation (IDF) averages around 425 million adults and there is a projected forecast of it rising to 700 million by 2045. As per IDF around 15.9 million of adult population are affected by DM as of 2017 with an overall regional prevalence of 3.1% with a estimated rise of up to 47 million approximating to 156% by the year 2045 [2]. The total number of patients of Undiagnosed Diabetes Mellitus (UDM) is higher in sub-Saharan Africa and is a leading contributor towards overall morbidity and mortality due to various infectious and non-infectious diseases in sub-Saharan Africa. An urgent control measure for earlier diagnosis and management of UDM is needed to avoid DM induced complications and deterioration in the quality of life. The disease burden in sub-Saharan Africa has increased due to lack of medical specialists, lack of awareness of health in the population, lack of organized structure for chronic disease care, shortage of paramedical staff, fewer tertiary care hospitals, and inappropriate health care information systems [3]. Moreover corona virus disease 2019 caused due to Severe Acute Respiratory Syndrome Corona Virus 2 (SARS COV 2) also referred as COVID-19 was declared as pandemic on March 11^th^ 2020 by the World Health Organization and has led to a devastating effect on health and economy of all countries globally including Africa [4]. The increased susceptibility to infections in patients of UDM may prove to be fatal and these patients may be at a higher risk for mortality in this unprecedented COVID-19 pandemic. These facts gave us an impetus to explore the challenges, risk and needed preventive measures in UDM patients in view of COVID-19 pandemic, hence we decided to analyse research data to explore the risk and the management of UDM patients contracting COVID-19 infections. Taking timely precautions and implementing preventive practices in UDM patients against COVID-19 will help preventing mortality and morbidity in these patients.

**Diabetes and undiagnosed diabetes mellitus in Africa:** the larger proportion of patients affected with DM in sub-Saharan Africa are in median age group of lesser than 50 years of age, and suffering from pancreatic beta cell secretary dysfunction and while western population have shown development of DM post 50 years of age and are Type II DM [3,5]. The magnitude and incidence of diabetes in sub-Saharan Africa is at a rise and the main concern is that many of the patients are undiagnosed and by the time they are identified they are already presenting with DM related complications. The pooled prevalence of UDM in African Community is around 5.3% and varies from as high of 18.1% in South Africa to Nigeria 4.6%, Tanzania 9%, Northern Sudan 5.9 %, Senegal 4.67% to quote a few and is attributed to lack of diagnostic facilities, poor health care system, lack of trained medics, shortage of paramedics and lack of health awareness in population [3]. As per global data of 2014 there are about 179.2 million UDM worldwide. The highest percentage of UDM patients are in Africa. Nearly 62.3% of the DM patients do not know that they are having diabetes while around 13.4 million had UDM. The early diagnosis and management are primary vital interventions to prevent the occurrence of complications and also to prevent deaths from diabetes in sub-Saharan Africa [5].

**COVID-19:** COVID-19 (SARS-CoV-2 Corona Virus) disease which has impacted the health of community globally manifests with symptoms such as fever, dry cough, myalgia, sore throat, dyspnoea, fatigue, vomiting, diarrhoea, and loss of taste and smell sensations. It has been observed that most of the patients of Covid-19 have a mild course of the disease but a few may progress to the stage of complications and develop acute respiratory distress syndrome (ARDS), sepsis, pneumonia and multiple organ failure [4]. The diagnostic method which is commonly being used is by processing the nasopharyngeal swab by Real Time Reverse Transcription Polymerase Chain Reaction [6]. The recommended guidelines for prevention of COVID-19 infection spread includes use of a face mask, regular hand wash with soap, social distancing. Social isolation is advocated if one has come in contact with COVID-19 positive case. The idea of work from home for employees & learn from home for students have been implemented in most of the countries.

**COVID-19 infection and high mortality risk in Africa:** as per the data analysis by predicted modelling by the World Health Organization, regional office for Africa, there can be about 83,000 to 1,90,000 deaths and around 29 million to 44 million people can get infected during the first year of the pandemic [7].

**COVID-19 in undiagnosed diabetic patient´s:** the patients of UDM are at higher risk of contracting COVID-19 infections. More over various epidemiological studies which have been conducted have recognized DM as the primary co-morbid condition linked with severe or lethal MERS-CoV infection. In an animal model study conducted on mice, it was observed that mice which were induced with DM by feeding them with high fat diet and then made susceptible to MERS-CoV (Middle East Respiratory Syndrome Corona Virus) by expressing DPP, had severe manifestations of COVID-19 with prolonged recovery time. The finding of this study was suggestive of dysregulation of immunological responses in MERS-CoV infection leading to severe and prolonged lung pathology in them [8]. These facts are of a great concern in patients of UDM contracting COVID-19 infections as they have chances of severe manifestation of pulmonary pathology and thereby high mortality risk as these UDM patients have usually developed diabetes associated complications by the time they get diagnosed. The patients of DM, hypertension and obesity are having significant risk for hospitalization and mortality with COVID-19 infections. The unholy interaction between DM and COVID-19 sets up a cascade of event in which COVID-19 produces critical dysglycemia and DM exacerbates severity of COVID-19. The cytokine storm in COVID-19 may induce insulin resistance and directly cause beta cell damage and this may lead to worsening dysglycemia in patient of DM having contracted COVID-19 infection [9]. In view of the increased mortality risk of DM with COVID-19, the undiagnosed DM patients may be one of the worst sufferers if they happen to contract COVID-19 infection. The higher percentage of UDM patients in Africa in the scenario of COVID-19 pandemic may also progress to morbid states with COVID-19 and may add up to the mortality rates.

**Safety measures for patients of undiagnosed diabetes mellitus against COVID 19 infection:** Africa´s disease burden especially of DM, HIV (Human Immunodeficiency Virus) and Tuberculosis leads to a heavy economic burden on the already strained health resources and economy in the region. Due to the COVID-19 pandemic the situation has worsened. In view of the increased severity of disease manifestation in patients of DM who contract COVID-19, it is necessary that a suitable advisory by health authorities should be released in Africa so that these patients of DM and UDM can be careful and alert towards prevention measures against COVID-19 infection as well as take adequate care for diabetes mellitus. The designed policy can be in lines as recommended by International Diabetes Federation and as practiced globally [2, 10]. Some guidelines are highlighted below: 1) All persons having a family history of DM, Obesity, hypertension must get screened for diabetes as those with UDM are at a potential risk of developing fulminating pulmonary pathology after contracting COVID-19 infections. 2) All patients of DM must maintain social distancing, use face mask, follow personal hygiene and adhere to isolation in case they come in contact with COVID-19 patients. 3) All patients of DM should have regular blood glucose monitoring and should take timely medication. 4) All patients of DM should have adequate supply of medication at home if required and avail home delivery services from pharmacies to avoid unnecessary exposure. 5) Encourage patients to consult doctors on phone & avail telemedicine service to avoid risk of contact with public. 6) Encourage patients to have adequate food to avoid hypoglycaemia so that need does not arise for a visit to the hospital and risk of exposure to COVID-19 in hospital set up. 7) Encourage patients to have adequate water intake in the hot summer environment. Adequate hydration and nutrition will boost up immunity. 8) Educate patients to keep themselves fit, by exercising at home, by brisk walking on a tread mill or aerobic exercises so that their blood glucose is controlled. This will also be helpful in patients of hypertension and obesity who are more prone to develop diabetes. 9) The patents of UDM with complications may develop more a severe manifestation of COVID-19 infection and need to be more cautious about adequate hydration, nutrition and exercise to enhance immunological response in this critical time of COVID-19 pandemic.

## Conclusion

The patients of diabetes mellitus and undiagnosed diabetes mellitus may contract COVID-19 in infection due to increased susceptibility of patient of diabetes to infections. The COVID-19 infection produces inflammatory response with cytokine storm and cytokines may induce insulin resistance and direct beta cell damage leading to worsening dysglycemia. A severe course and delayed recovery from SARS CoV infection in patients of DM is a lesson we learnt from the past; and now COVID-19 is similarly showing severe manifestations with increased risk of mortality in diabetics. Hence the patients of DM as well as the high-risk group of UDM should practice safety measures of isolation, social distancing and personal hygiene to prevent the morbidity and mortality. The measures for containment need to be practiced in sub-Saharan Africa for slowing down the rate of transmission and must include contact tracing, social distancing, isolation and strictly practicing personal hygiene.
